# Tristetraprolin affects invasion‐associated genes expression and cell motility in triple‐negative breast cancer model

**DOI:** 10.1002/cm.21934

**Published:** 2024-09-25

**Authors:** Anastasiia Hubiernatorova, Josef Novak, Michaela Vaskovicova, David Sekac, Serhii Kropyvko, Zdenek Hodny

**Affiliations:** ^1^ Department of Functional Genomics Institute of Molecular Biology and Genetics NAS of Ukraine Kyiv Ukraine; ^2^ Laboratory of Cell Regeneration and Plasticity Institute of Animal Physiology and Genetics of the Czech Academy of Sciences Libechov Czech Republic; ^3^ Laboratory of Genome Integrity Institute of Molecular Genetics of the Czech Academy of Sciences Prague Czech Republic; ^4^ Department of Cell Biology, Faculty of Science Charles University Prague Czech Republic; ^5^ Laboratory of DNA Integrity Institute of Animal Physiology and Genetics of the Czech Academy of Sciences Libechov Czech Republic

**Keywords:** breast cancer, doxorubicin, invasion, motility, tristetraprolin

## Abstract

Tristetraprolin (TTP) is an RNA‐binding protein that negatively regulates its target mRNAs and has been shown to inhibit tumor progression and invasion. Tumor invasion requires precise regulation of cytoskeletal components, and dysregulation of cytoskeleton‐associated genes can significantly alter cell motility and invasive capability. Several genes, including *SH3PXD2A, SH3PXD2B*, *CTTN*, *WIPF1*, and *WASL*, are crucial components of the cytoskeleton reorganization machinery and are essential for adequate cell motility. These genes are also involved in invasion processes, with *SH3PXD2A*, *SH3PXD2B*, *WIPF1*, and *CTTN* being key components of invadopodia—specialized structures that facilitate invasion. However, the regulation of these genes is not well understood. This study demonstrates that ectopic expression of TTP in MDA‐MB‐231 cells leads to decreased mRNA levels of *CTTN* and *SH3PXD2A*, as well as defects in cell motility and actin filament organization. Additionally, doxorubicin significantly increases TTP expression and reduces the mRNA levels of cytoskeleton‐associated genes, enhancing our understanding of how doxorubicin may affect the transcriptional profile of cells. However, doxorubicin affects target mRNAs differently than TTP ectopic expression, suggesting it may not be the primary mechanism of doxorubicin in breast cancer (BC) treatment. High TTP expression is considered as a positive prognostic marker in multiple cancers, including BC. Given that doxorubicin is a commonly used drug for treating triple‐negative BC, using TTP as a prognostic marker in this cohort of patients might be limited since it might be challenging to understand if high TTP expression occurred due to the favorable physiological state of the patient or as a consequence of treatment.

## 1 INTRODUCTION

Breast cancer (BC) is one of the most common malignancies in women; according to the World Health Organization, in 2020, it was diagnosed in 2.3 million patients worldwide and caused 685,000 deaths (Arnold et al., 2022). It affects the individuals diagnosed and has far‐reaching effects on families and communities. Despite numerous studies conducted to reveal the mechanisms of BC initiation and progression, further investigations are still needed to improve prevention, detection, and treatment strategies. Since metastasis is one of the most common complications in malignancies, leading to tumor spreading, organ failure, and death, it is important to investigate mechanisms of metastasis formation.

Post‐transcriptional regulation of gene expression is a well‐orchestrated mechanism that is realized by different players, such as non‐coding RNAs and RNA‐binding proteins (RBPs). In healthy cells, the expression of survival‐associated genes is tightly controlled and maintained at normal levels by different mechanisms, including post‐transcriptional regulation. However, tumor cells could overcome this control and show abnormal levels and stability of pro‐survival genes. One of the possible ways for malignant cells to avoid post‐transcriptional regulation is to alter the expression of tumor suppressors, such as tristetraprolin (TTP).

### 1.1 TTP protein family and its role in cancer

TTP (also known as ZFP36, IS11b, cMG1, ERF1, BRF1, and Berg36) is a 326 amino acid protein that belongs to the ZFP36 protein family. ZFP36 family members are RBPs that bind to AU‐rich elements (AREs) of their target transcripts and promote their degradation, which can lead to changes in gene expression. In mammals, this family includes three members: ZFP36, ZFP36L1, and ZFP36L2 (Blackshear, 2002). One more member, ZFP36L3, is exclusively expressed in rodents and is placenta specific (Blackshear et al., 2005). ZFP36 family members possess three domains: nuclear export signal at N‐terminus, tandem CCCH zinc finger domain, and CNOT‐binding domain at C‐terminus, although specific structural details of ZFP36 proteins, including the number and arrangement of zinc finger domains or other motifs, can vary within the family and contribute to their distinct functions and target specificity (Johnson & Blackwell, 2002; Kafasla et al., 2014). TTP exhibits sequence‐specific binding to AREs located in the 3′ untranslated regions (3′UTRs) of its target transcripts and facilitates mRNA decay through various mechanisms, including deadenylation, suppression of polyadenylation, and via enhancing of 5′–3′ decay by recruiting of decapping machinery (Fabian et al., 2013; Lai et al., 1999; Su et al., 2012).

As an ARE‐binding protein, TTP is involved in the regulation of multiple pathways via the regulation of target ARE‐containing RNAs, which are present in more than 8% of cellular transcripts (Bakheet et al., 2006). It plays a critical role in post‐transcriptional gene regulation and is involved in various biological processes, including immune response, cell cycle control, and maintaining cellular homeostasis. Its ability to selectively target and destabilize specific mRNAs makes it an important regulator of gene expression, and its dysregulation has been implicated in various diseases, including inflammatory disorders and cancer (Rodríguez‐Gómez et al., 2021). TTPs expression level is significantly decreased in various cancer types, such as bladder, breast, colon, prostate, hepatic, cervical, and other cancers. Thus, TTP is considered to be an antitumorigenic protein (Saini et al., 2020).

Since TTP is extensively studied as a tumor suppressor, it is often considered a positive prognostic marker for various malignancies: its downregulation or functional defects are frequently associated with tumor progression and poor prognosis (Brooks & Blackshear, 2013; Essafi‐Benkhadir et al., 2007; Rounbehler et al., 2018). In BC, it is shown to be significantly downregulated, and its expression conversely correlated with tumor aggressiveness and metastatic potential (Brennan et al., 2009; Griseri et al., 2011). Moreover, BC patients with low TTP expression levels were shown to have poorer survival rates and more aggressive tumors, thus indicating TTP can be a promising candidate as a clinical biomarker (Fallahi et al., 2014). However, we previously investigated the expression of TTP in different subtypes of BC and showed that it varies between subtypes. Although our data also show that the overall and disease‐free survival rate is indeed higher in patients with high TTP expression, if we divide the data by BC subtypes high TTP expression may correlate with both increased and decreased survival rates compared to the low expression cohort (Serhii Kropyvko et al., 2023).

TTP is recognized as a tumor‐suppressive protein based on numerous studies demonstrating its capacity to reduce the expression of genes linked to cancer progression, many of which have specifically documented a decrease in the expression of TTP proteins in diverse cancer types (Saini et al., 2020). For instance, Brennan and collaborators showed a significant decrease of TTP mRNA levels in a wide range of tumor types, including advanced breast and prostate cancers. The restoration of TTP expression in aggressive tumor cell lines effectively inhibited three critical tumorigenic phenotypes: cell proliferation, resistance to pro‐apoptotic signals, and the expression of VEGF mRNA (Brennan et al., 2009). Moreover, the authors showed that a decrease in TTP expression was a negative prognostic marker in BC since patients with low tumor TTP expression were more likely to exhibit higher tumor grade, VEGF expression, and mortality from recurrent disease.

One interesting study on TTP role in hepatic cancer conducted on mice suggests that TTP plays a dual role in liver cancer development, promoting tumor initiation but potentially inhibiting progression to malignancy (Dolicka et al., 2021). While deletion of TTP in the liver did not cause spontaneous tumor development or liver damage in aged mice, the carcinogenic agent diethylnitrosamine led to the development of liver tumors, including hepatocellular carcinoma. However, mice lacking TTP showed a significant reduction in tumor formation compared to control mice. Despite this reduction, the tumors in TTP‐deficient mice progressed to hepatocellular carcinoma at a higher rate.

There is evidence that TTP may reduce migration and invasion potential in different cancer types via regulating multiple targets. For example, overexpression of TTP inhibited migration and invasion of gastric carcinoma cells through the regulation of IL‐33 and in glioma by regulation of IL‐13 and uPAR (Deng et al., 2016; Ryu et al., 2015; Zeng et al., 2016). Inhibition of TTP led to an increase of MMP9, MMP6, and IL‐6 in head and neck carcinoma. MMP9 and MMP6 are metalloproteinases important for matrix degradation, which is required for sufficient invasion, and IL‐6 is associated with tumor progression and invasion via regulating proliferation, migration, invasion, and survival of cancer cells. TTP inhibition yielded increased levels of these targets, and miRNA‐induced depletion of TTP resulted in increased tumor growth in pancreatic cancer (Van Tubergen et al., 2013).

### 1.2 Cytoskeleton in motility and invasion

Metastatic tumor behavior depends on tumor cell overall motility and ability to detach from the primary tumor, invade the extracellular matrix, and subsequently enter the blood or lymphatic vessels to spread to lymph nodes and distant tissues. In BC, it has been shown that cancer cells can disseminate not only from primary tumors and metastatic lymph nodes but also from lung metastases highlighting the ongoing importance of investigation of invasion mechanisms (Borriello et al., 2021). Invadopodia, a specialized type of invadosome, play a crucial role in integrating signals from the tumor microenvironment to facilitate tumor cell invasion and spread. Recent advancements have revealed how tumor cells manage the flexibility required for invadopodia to form and operate effectively across various in vivo microenvironments encountered during dissemination (Linder et al., 2023).

These processes require multiple proteins and protein complexes, and one of the most important is actin reorganization machinery. The dynamics of the actin cytoskeleton are tightly regulated by intracellular signaling pathways involved in invasion. Signaling molecules such as Rho GTPases, Src kinase, and phosphoinositide 3‐kinase regulate actin polymerization, cell adhesion, and motility during invasion (Carpenter, 2000). Dysregulation of these signaling pathways can promote invasive behavior in cancer cells. Cytoskeleton reorganization itself is a complex process that allows multiple cellular events, such as spatial organization of cellular components, division, exo‐ and endo‐cytosis, acquisition and maintenance of specific morphology, cell motility, migration, and invasion. Realizing such events requires precise and orchestrated regulation of cytoskeletal components, including actin, intermediate filaments, and microtubules (Fletcher & Mullins, 2010). Dynamic reorganization of actin filaments facilitates directed movements and requires the participation of different factors, such as actin nucleation, elongation and polymerization factors, actin stabilization and destabilization factors, as well as scaffold proteins to assemble multiprotein complexes, and GTPases to activate/inactivate components, involved in the movement (S. H. Lee & Dominguez, 2010). In order to move, cells form specific actin‐enriched protrusions called podosomes, or “cellular feet.” In cancer cells, podosomes can degrade the extracellular matrix, enabling extravasation. These specialized podosomes are called invadopodia and may be distinguished from normal podosomes via the presence of specific proteins.

Actin nucleation promoting factors (NPFs) are required to enable the branching activity of the Arp2/3 complex, which generates actin filaments, and are represented by two classes: NPFs Class I, which share the Arp2/3 complex and actin monomer interaction surfaces in close proximity and NPFs Class II, which can interact with the Arp2/3 complex and to bind actin filaments (Alekhina et al., 2017; Suetsugu, 2013; Weaver et al., 2001). WASP protein superfamily are well‐studied Type I NPFs, which promote rapid polymerization of filaments under the cellular membrane, an essential step for diverse physiological and pathophysiological processes, including invasion. N‐WASP member (encoded by *WASL* gene) recruits actin monomers to the Arp2/3 complex, stimulating its activity to form a stable actin filament nucleus (Helgeson & Nolen, 2013; Rohatgi et al., 2000). WIP (WASP‐interacting protein encoded by the *WIPF1* gene) interacts with N‐WASP and regulates its localization, stability, and activity (Antón & Jones, 2006; García et al., 2012). These proteins together contribute to the formation and effective operation of invadopodia and podosomes, and their dysregulation has been associated with cancer progression and metastasis in several cancer types, including BC (García et al., 2012). Another NPF, cortactin, is encoded by the *CTTN* gene and belongs to NPF Class II. It is required for branched filaments formation and, together with WIP and N‐WASP, is required for invadopodia formation (Ayala et al., 2008; García et al., 2012; Siar et al., 2016). Cortactin was shown to enhance N‐WASP activation of Arp2/3 complex and inhibit actin filaments debranching, subsequently promoting cell motility (Bryce et al., 2005; Weaver et al., 2001). N‐WASP and cortactin are shown to be essential components of invadopodia, together with scaffold protein TKS5, also known as *SH3PXD2A*. TKS5 is a large scaffold protein interacting with cortactin and N‐WASP to promote actin polymerization and reorganization within podosomes and invadopodia. It is localized exclusively in these structures (Buschman et al., 2009; Oikawa et al., 2008). Together with one more TKS protein, TKS4 (*SH3PXD2B*), it is involved in epidermal growth factor (EGFR) signaling, serving as a platform to assemble signaling machinery, promoting cellular migration and spreading (Bögel et al., 2012; S. V. Kropyvko, 2015; Lányi et al., 2011). Both TKS4 and TKS5 are involved in EGFR signaling, as well as cortactin. Once bound to its ligands, EGFR activates downstream pathways regulating actin cytoskeleton dynamics (Chen et al., 1994). TKS5 is a key scaffold protein that localizes to invadopodia and is essential for their assembly and function. It interacts with other invadopodia‐associated proteins, such as cortactin and N‐WASP, to regulate actin polymerization, membrane protrusion, and extracellular matrix degradation at invadopodia sites (Seals et al., 2005; Stylli et al., 2009). It promotes cancer cell migration and invasion in vitro and in vivo, and its overexpression correlates with increased invadopodia formation, matrix degradation, and metastatic potential in various cancer cell lines and animal models (Blouw et al., 2008; Oser et al., 2010). EGF‐activated cells possess elevated levels of PI(3,4,5)P_3_, and its dephosphorylation leads to the formation of PI(3,4)P_2_, which in turn recruits TKS5 to the plasma membrane and nucleates invadopodia and podosomes (Ben‐Chetrit et al., 2015). Downregulation of TKS5 may lead to insufficient formation of complexes required for invadopodia and podosome formation, resulting in decreased invasion and migration and insufficient signal transmission from EGFR.

### 1.3 Doxorubicin in brief

Doxorubicin is a widely used chemotherapeutic drug that is utilized for the treatment of different types of cancer, such as breast, prostate, uterus, ovary, bile duct, esophagus, liver, and others (Carvalho et al., 2009). It is known to intercalate DNA, resulting in the uncoiling of the double helix, subsequent inhibition of DNA, RNA, and protein synthesis, and death of rapidly dividing cells. It is one of the most common therapeutics used in BC and appears to be one of the most important for patients with triple‐negative breast cancer (TNBC), characterized by the absence of estrogen, progesterone, and HER2/neu receptors, and thus unavailable for hormonal therapy (Minotti et al., 2004; Nicoletto & Ofner, 2022).

Lately, doxorubicin has been extensively investigated in the context of altered gene expression associated with its cardiotoxicity and mechanisms of doxorubicin resistance. However, only little is known about how doxorubicin affects the expression of cytoskeleton‐associated genes. DNA damage caused by doxorubicin (DXR) leads to the activation of ATM kinase, which has numerous targets, and cell response in the context of the cytoskeleton may be prominent depending on the dynamic interplay between ATM‐activated pathways. Moreover, the effect of DXR may significantly vary depending on p53 status and lead to TNFα‐induced NF‐κB activation (Banno et al., 2004). For instance, DXR treatment in p53‐mutated MDA‐MB‐231 cells activated NF‐κB‐driven gene signature, modulating genes related to invasion, metastasis, and chemoresistance. This pattern was also observed in other cell lines and primary human breast tumors; restoring wild‐type p53 in these cells impaired the DXR‐induced NF‐κB‐driven transcription (Dalmases et al., 2014). There is also evidence that DXR‐induced NF‐κB promotes BC cell migration and metastasis via inducing CXCR4 (Helbig et al., 2003). On the other hand, there is a study showing that although DXR induces NF‐κB signaling and produces active NF‐κB complexes, they are deficient in phosphorylation and acetylation, which results in repressed constitutive‐ and cytokine‐induced NF‐κB‐dependent transcription (Ho et al., 2005).

To summarize, despite numerous studies investigating the effects of TTP on invasion potential in different cancer types, our understanding of how TTP affects cytoskeletal components remains limited. In this study, we attempted to examine whether ectopic TTP expression could affect the levels of cytoskeleton‐related genes and consequently affect cell motility. Furthermore, we investigated the potential effects of doxorubicin treatment on TTP expression and mRNA levels of target genes associated with the cytoskeleton.

## 2 RESULTS

### 2.1 In silico analysis of 3′UTRs of target cytoskeleton‐associated mRNAs

First, we analyzed 3′UTRs of 33 target genes (54 transcript variants), important for cytoskeleton reorganization and invadopodia formation to identify regulatory elements by in silico analysis (detailed information is provided in Table S1; a summary is presented in Table 1).

**TABLE 1:** General information on target mRNAs analysis.

		Bioinformatic resource		
3′UTR length range 200–11,561 nt		Scan for motifs	RegRNA 2.0	Both
Regulatory element	Musashi‐binding element	52	41	36
	ARE (AU‐rich elements)	24	18	14
	K‐box	17	12	10
	GU‐rich element	13	7	6
	Brd‐box	5	9	5
	GY‐Box	8	7	3
	C‐Rich stability element	6	0	0
	Grb‐box	1	1	0
	Pumilio‐binding element	12	0	0
	SECIS1	0	3	0
	UNR‐binding site	0	7	0

The analysis revealed two predominant regulatory elements: Musashi‐binding element (MBE) and ARE. MBE serves as a binding site for Musashi proteins, which are frequently up‐regulated in various malignancies and are considered tumor‐promoting via repressing target mRNAs, encoding cell cycle inhibitory proteins (reviewed by Kudinov et al., 2017). Since our idea was to find the regulatory element involved in the repression of invasion‐promoting transcripts, we excluded MBEs from further investigation. Instead, we focused on ARE, another common regulatory element. AREs are known to be important determinants of mRNA stability, as well as being binding sites for TTP, so further, we estimated the probability of binding of TTP with ARE‐containing transcripts involved in invadopodia formation, using publicly available online resources RPISeq and omiXcore. The first resource allows to estimate the global probability of protein–RNA binding, and the second one allows to estimate such probability locally (provides information on the localization of binding signals along the entire length of the target RNA and estimates the probability separately for each signal). Moreover, the number of AREs is important for the successful binding of RNA‐binding protein with its target transcript, and AREScore, which reflects the number of predicted AREs within a particular RNA, was also estimated. The values of all listed parameters are shown in Table 2.

**TABLE 2:** Estimation of interaction probability between tristetraprolin (TTP) and target transcripts.

Transcript	Probability (omiXcore)	Probability (RPISeq)	AREScore
*SH3PXD2B*	0.21	0.90	5.00
*WASL*	0.13	0.80	16.06
*SH3PXD2A*	0.87	0.85	18.35
*WIPF1*	0.69	0.95	11.03
*CTTN*	0.3	0.80	2.03

As might be seen from Table 2, the omiXcore algorithm revealed a high binding probability for *SH3PXD2A* and *WIPF1*. In contrast, the RPISeq algorithm revealed a high binding probability for all the targets. Therefore, we chose to conduct further research on all the targets listed in Table 2.

### 2.2 TTP ectopic expression affects levels of cytoskeleton‐associated mRNAs

**FIGURE 1:**
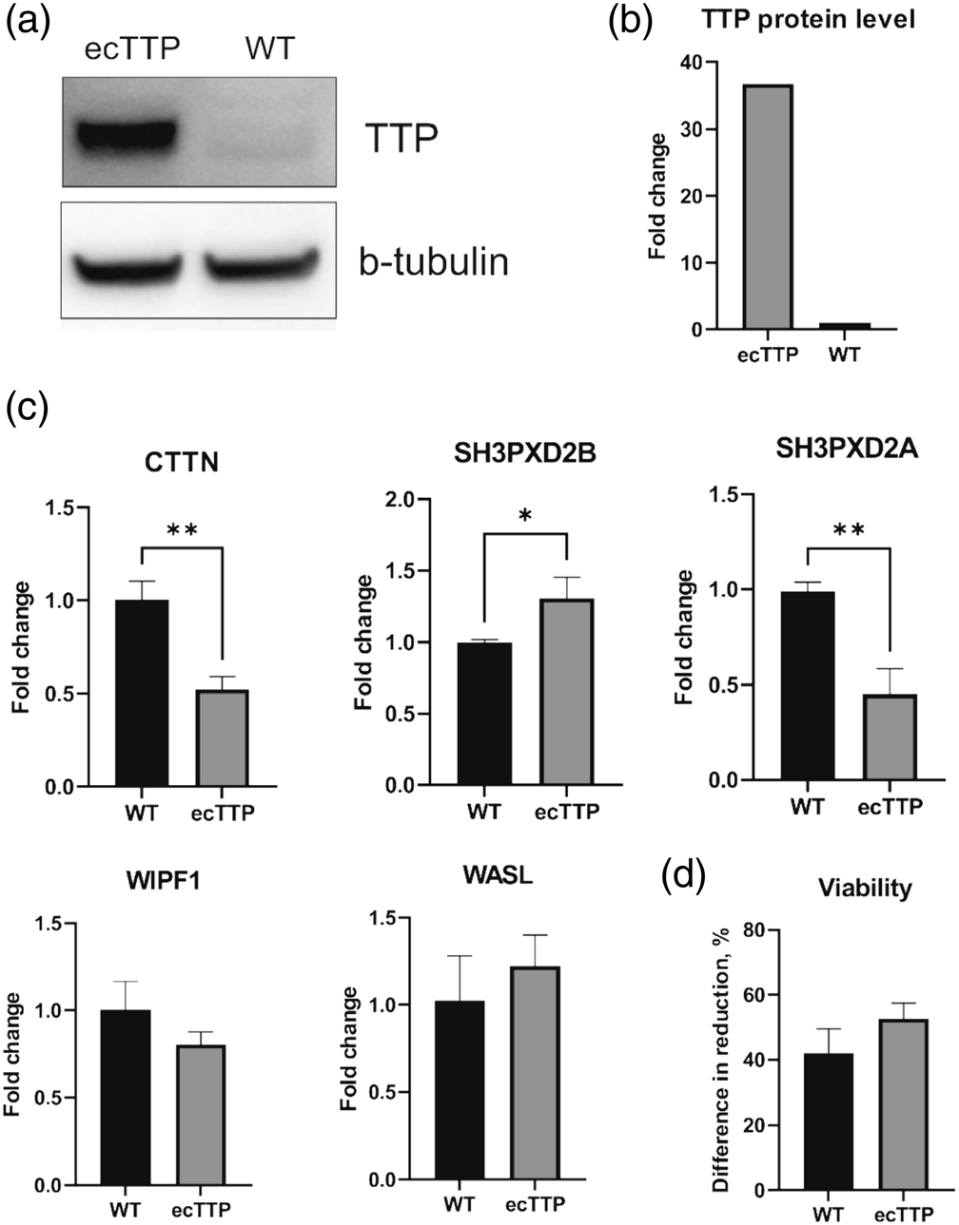
Analysis of TTP and target mRNAs' expression and viability of stable transfectants. (a,b) Western blotting analysis was performed to ensure the expression of TTP in stable transfectants. (c) Effects of ectopic expression of TTP on target mRNA levels (*n* = 3). (d) Effects of ectopic expression of TTP on the viability of stable transfectants (*n* = 5). Graphs show mean ± standard deviation. *p < 0.05; **p < 0.01; ***p < 0.001; ****p < 0.0001. ecTTP, stable transfectants with ectopic TTP expression; TTP, tristetraprolin; WT, wild‐type cells.

To investigate whether TTP may affect expression levels of target invasion‐associated genes, we generated a stable invasive MDA‐MB‐231 BC cell line, which corresponds to TNBC, with ectopic expression of TTP. TTP protein levels in stable transfectants were significantly increased compared to normal cells (fold change 36; Figure 1a,b). TTP is an early‐response gene and is expressed at a low basal level. Usually, its expression dramatically increases as a response to different stimuli. However, after the stimulus is removed, it returns to its normal level (Cao, 2024; Sachidanandan et al., 2002). Thus, its constant high expression may lead to changes in survival. We performed the Alamar Blue assay (Figure 1d) to estimate the in vitro viability of stable transfectants, and the results showed that the viability of cells ectopically expressing TTP did not differ from the wild‐type cells. To investigate further if TTP may affect the expression of cytoskeleton‐associated genes, we analyzed their mRNA levels via RT‐qPCR (Figure 1c). While TTP overexpression did not change mRNA levels of *WIPF1* and *WASL*, it significantly changed the mRNA levels of *CTTN*, *SH3PXD2A*, and *SH3PXD2B. CTTN* and *SH3PXD2A* mRNA levels were nearly 2‐fold decreased, and *SH3PXD2B* showed a 1.3‐fold increase.

### 2.3 TTP ectopic expression affects cell motility, morphology, and invasion capacity

**FIGURE 2:**
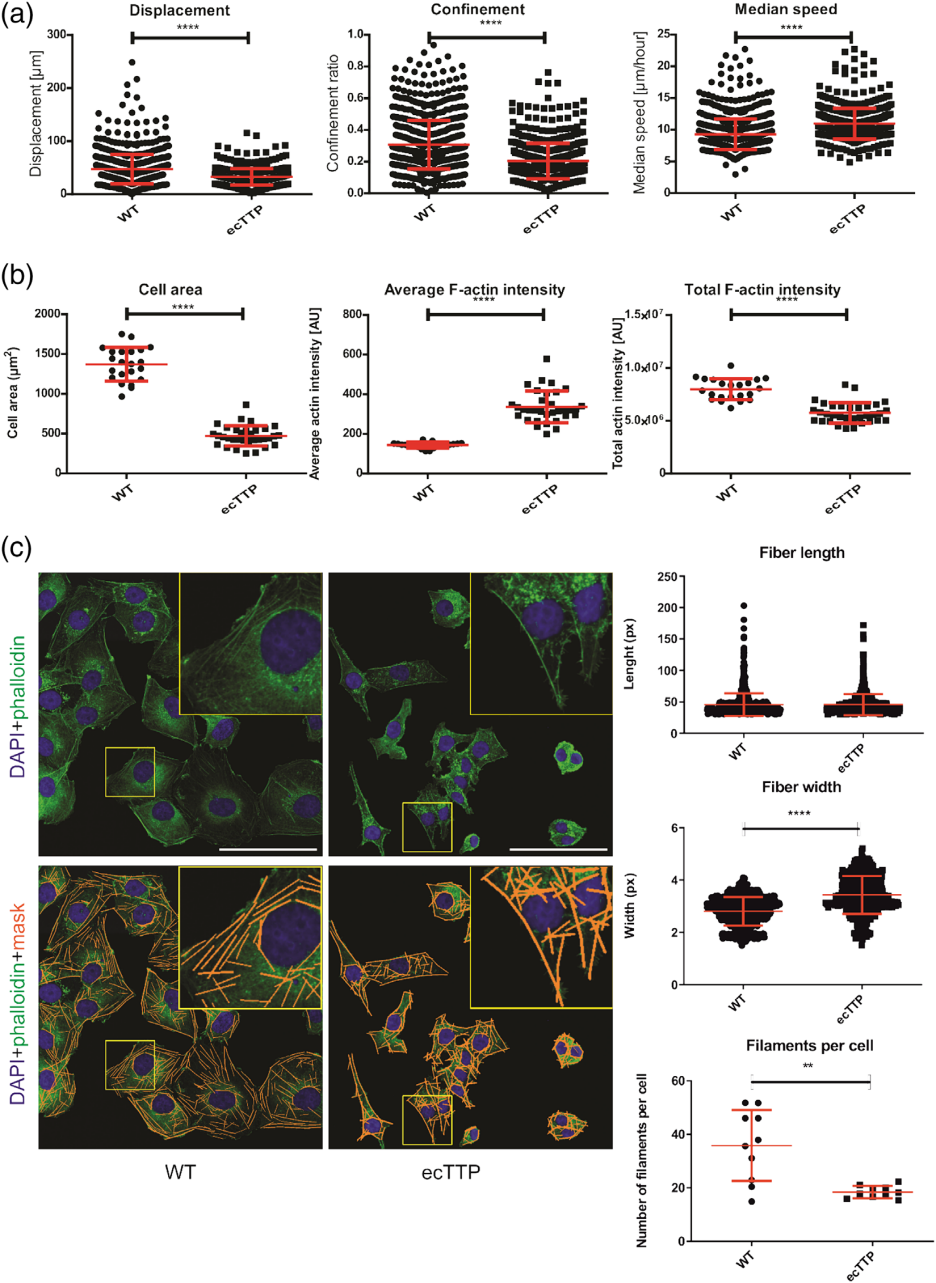
Changes of cellular motility in MDA‐MB‐231 cells ectopically expressing TTP. (a) Effects of ectopic TTP expression on cells' displacement, confinement ratio, and speed; *n* = 14; (b) Effects of ectopic expression of TTP on cellular morphology and intensity of F‐actin staining; (c) representative images of MDA‐MB‐231 ectopically expressing TTP and the control cells, stained by phalloidin and DAPI (top) and with mask of actin fibers (orange) detected by FilamentSensor 2.0, bottom. Scale bar, 100 μm; *n* = 10. Insets and yellow rectangles show typical 50 × 50 μm regions. Graphs show significant differences in the length and width of actin fiber masks and the number of filaments as found by FilamentSensor 2.0. Graphs show mean ± standard deviation; *n* = 10; *p < 0.05; **p < 0.01; ***p < 0.001; ****p < 0.0001. TTP, tristetraprolin.

Since previous findings showed that ectopic expression of TTP resulted in decreased expression of *SH3PXD2A* and *CTTN* mRNAs, we suggested that it may result in impaired motility. To analyze cell motility, we followed the non‐directed movement of cells in 2D by tracking nuclei of wild‐type MDA‐MB‐231 cells and cells with ectopic expression of TTP labeled by SiR‐DNA and SPY555‐actin and observing them every 15 min for 24 h (for representative image of the last time point of the tracked cells, see Figure S1A and Video S1 for a representative video of the tracked cells). Ectopic expression of TTP significantly decreased cell displacement (distance from start to end point) and decreased directionality of movement, as indicated by the decrease in confinement ratio. However, the speed of the nuclei movement in cells ectopically expressing TTP significantly increased, indicating more frequent shifts of centers of the mass of the nuclei despite the lack of effective movement of the cells (Figure 2a).

Since actin staining in the live‐cell imaging (Figure S1A) suggested changes in the actin cytoskeleton, we fixed the cells, stained them with phalloidin and obtained higher resolution confocal images. Individual cells in the images were segmented by the CellPose algorithm, and morphological characteristics and intensity of actin staining were quantified by ImageJ (Figure 2b; for representative images of the segmentation, see Figure S1B). Cells expressing TTP were significantly smaller (fold change 3.04), although their shape (circularity and aspect ratio; Figure S1C) did not change. Cells with ectopic TTP expression showed increased average F‐actin signal, indicating denser F‐actin staining of the cells. However, the F‐actin signal integrated per cell revealed overall lower F‐actin intensity, which may reflect decrease in total cell size, but may also indicate decreased actin polymerization capacity.

To further analyze actin organization, the images were analyzed by FilamentSensor 2.0 (Hauke et al., 2023), and individual F‐actin fibers were analyzed (Figure 2c). Surprisingly, there was no difference in the length of F‐actin fibers, but identified fibers were significantly thicker and less abundant in cells overexpressing TTP. Upon closer inspection (see insets in Figure 2c), TTP overexpression induced stronger actin staining in the cortical region of the cells but significantly reduced number of filaments per cell.

**FIGURE 3:**
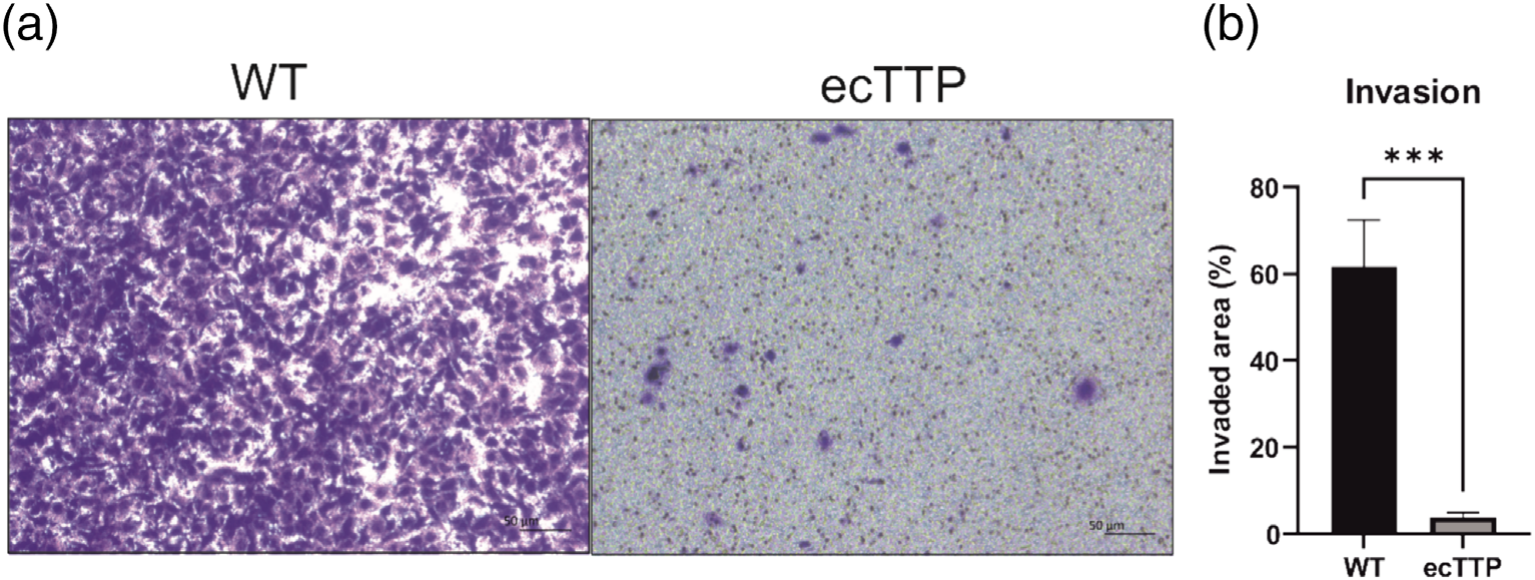
Invasion capacity of MDA‐MB‐231 cells with ectopic TTP expression. (a) Representative images of transwell invasion assay. (b) Invasion capacity of the cells by invaded area; WT MDA‐MB‐231 cells; *n* = 6. Graph shows mean ± standard deviation. *p < 0.05; **p < 0.01; ***p < 0.001; ****p < 0.0001. ecTTP, the cells with ectopic TTP expression; TTP, tristetraprolin; WT, wild‐type cells.

Regarding the results described above, we suggested TTP ectopic expression may not only affect motility and morphology, but also invasion capacity. To check this, we performed a transwell invasion assay (Figures 3a,b and S2A–D). The results showed a dramatic decrease in area invaded by the cells with ectopic TTP expression compared to wild‐type cells, indicating that ectopic TTP expression decreases the invasion capacity of the cells, which in some part might be a consequence of their decreased motility.

### 2.4 Doxorubicin increases expression of TTP in MDA‐MB‐231 cells and affects mRNA levels of *CTTN* , *SH3PXD2A* , *SH3PXD2B* , *WIPF1* , and *WASL*

**FIGURE 4:**
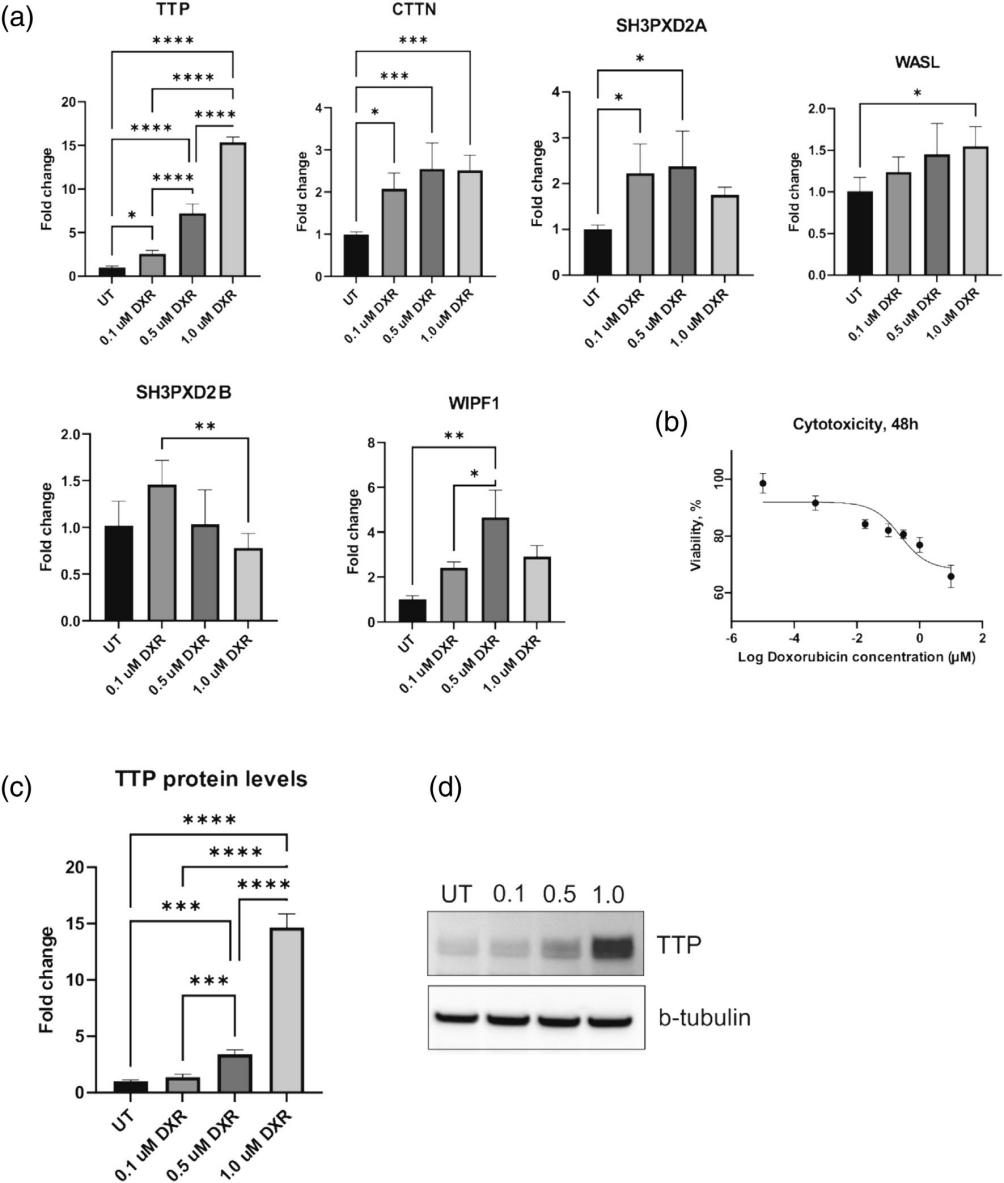
Effects of doxorubicin on target genes expression and cell viability. (a) mRNA levels of TTP, CTTN, SH3PXD2A, WASL, SH3PXD2B, and WIPF1 in control and doxorubicin‐treated cells; *n* = 6. (b) Cytotoxicity of doxorubicin for MDA‐MB‐231 cells after 48 h of treatment; (c) protein levels of TTP under doxorubicin treatment; *n* = 6. (d) Representative western blot of TTP protein levels under doxorubicin treatment. Graphs show mean ± standard deviation. Concentration of doxorubicin: 0.1, 0.5, and 1.0 μM. *p < 0.05; **p < 0.01; ***p < 0.001; ****p < 0.0001. TTP, tristetraprolin; UT, untreated cells.

To investigate if doxorubicin affects the expression of TTP, wild‐type MDA‐MB‐231 cells were treated with 0.1, 0.5, and 1.0 μM of DXR, which are considered to be clinically relevant concentrations (Nicoletto & Ofner, 2022). IC_50_ = 0.19 and IC_90_ = 2.71 μM were calculated prior to treatment (Figure 4b). As shown in Figure 4a, even a low concentration of 0.1 μM led to a nearly 2.5‐fold change of TTP mRNA level compared to untreated cells, with subsequent increase of 7 and 15 times under treatment with 0.5 μM, and 1.0 μM of doxorubicin, respectively. Western blotting analysis (Figure 4c,d) showed that DXR significantly increases TTP protein levels in a dose‐dependent manner. Increasing concentrations of DXR led to nearly 1.5‐, 3.5‐, and 15‐fold change in TTP protein level, not only supporting the results obtained from RT‐qPCR but also showing significant correlation between TTP mRNA and protein levels (Spearman correlation coefficient *r* = 0.9).

Considering impact of TTP on target cytoskeletal mRNA levels in the transformed cells and the described above results on DXR‐inducing TTP expression, we hypothesized that DXR may also affect the target cytoskeletal mRNA levels. To check this, we performed RT‐qPCR analysis on the cells treated with the above concentrations of DXR (Figure 4a). Interestingly, it not only induced TTP expression but also induced the levels of all target mRNAs, but not in the way expected. *SH3PXD2B* initially showed a nonsignificant increase at 0.1 μM concentration but subsequently returned to the initial level. In contrast, *WASL* mRNA levels stayed stable under 0.1 and 0.5 μM concentrations and increased only at 1.0 μM. At the same time, DXR significantly increased the mRNA level of *CTTN* compared to untreated cells, which plateau already at 0.1 μM and showed no further increase with increased concentration of DXR. Interestingly, *SH3PXD2A* and *WIPF1* showed noticeable variability in mRNA levels, especially at 0.5 μM concentration. We suggest that the absence of the expected levels of target mRNAs observed in the cells with TTP ectopic expression can be explained by the fact that DXR causes massive DNA damage and activates multiple signaling pathways, which may obscure the effects of TTP. Moreover, the fold increase of DXR‐induced TTP is twice as low as observed in the cells with ectopic TTP expression. One of the other possible explanations is that the TTP level in DXR‐treated cells might not be high enough. Nevertheless, we suggest that both genotoxic effect of DXR and lower TTP expression compared to ecTTP cells contributed to the observed phenotype.

### 2.5 Doxorubicin affects cell motility and morphology

**FIGURE 5:**
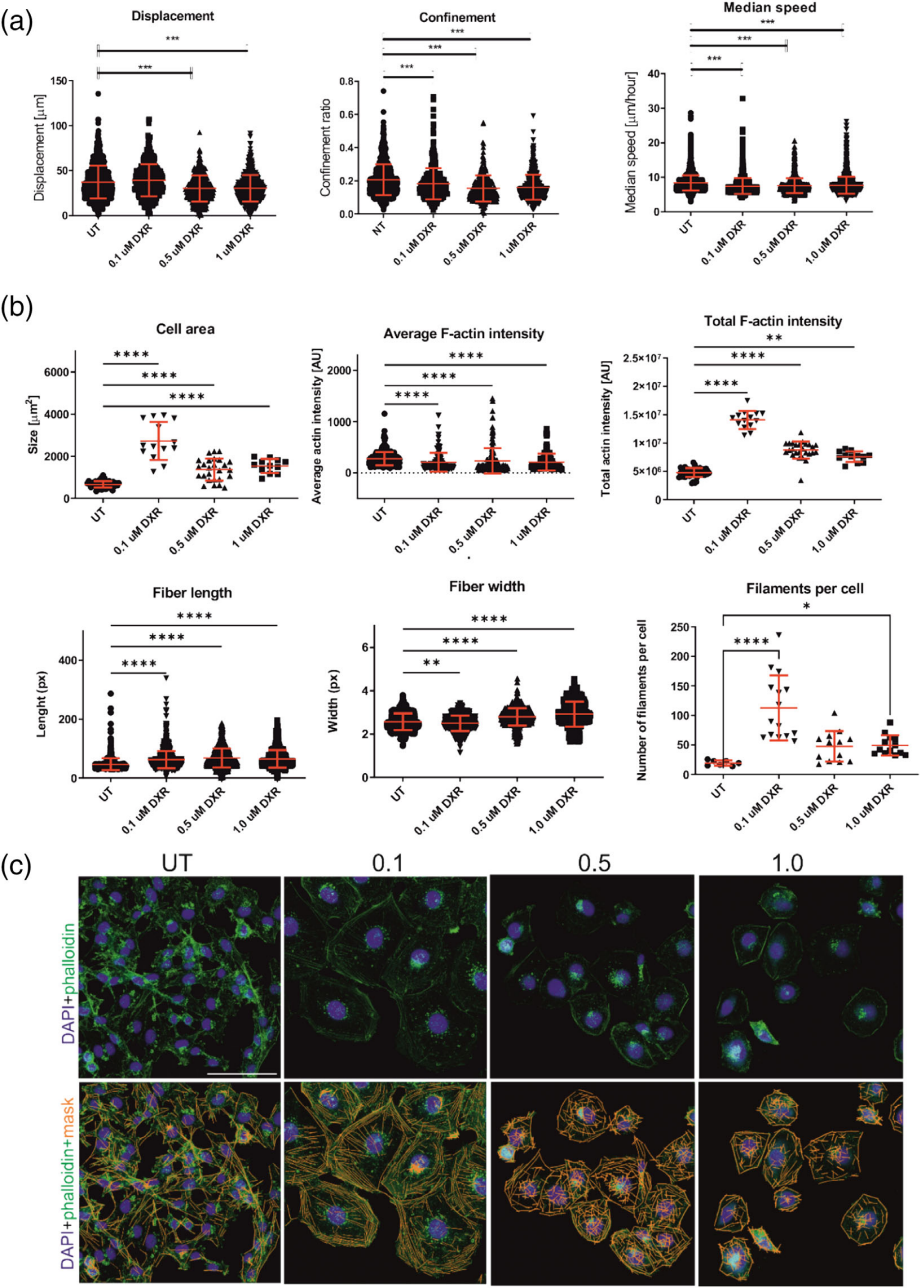
Changes of cellular motility in MDA‐MB‐231 cells exposed to DXR for 48 h. (a) Changes of cellular motility after exposing to different DXR concentrations; *n* = 10; (b) analysis of cellular morphology and actin filaments; *n* = 10; (c) changes of cellular morphology visualized by phalloidin and DAPI staining (top) and with mask of actin fibers (orange) detected by FilamentSensor 2.0, bottom. Scale bar, 100 μm. Graphs show mean ± standard deviation. *p < 0.05; **p < 0.01; ***p < 0.001; ****p < 0.0001. DXR, doxorubicin.

Since DXR treatment led to changes in both expressions of TTP and target cytoskeletal mRNAs, we tracked the wild‐type cells 48‐h post‐doxorubicin treatment to investigate whether DXR can affect cellular motility and morphology (Figures 5a and S3A, upper panel). The analysis showed that although 0.1 μM concentration of DXR did not lead to any changes in displacement, it decreased under the treatment with 0.5 and 1.0 μM of DXR. However, starting from the lowest concentration, the cells showed a decrease in confinement ratio and median speed.

We also investigated the effects of DXR treatment on cellular morphology. Our data showed a dose‐dependent effect on cellular morphology and actin filaments appearance (Figures 5b,c, S3A, bottom panel, and S3B). Interestingly, a low concentration of 0.1 μM led to a dramatic increase (fold change 3.7) in the cell area and a lesser increase in higher concentrations, accompanied by an increase in total F‐actin levels, which also peaked at 0.1 μM DXR. Despite the increase in total F‐actin level, the relative intensity of F‐actin staining slightly decreased. In 0.5 and 1 μM DXR, the width of actin filaments increased, indicating their bundling; in 0.1 μM DXR, the width of filaments slightly but significantly decreased. The length of individual actin filaments was only slightly affected. However, a change in area corresponded to a change in the number of detected actin filaments per cell.

Note that IC_50_ = 0.19 μM and IC_90_ = 2.71 μM of DXR were estimated prior to treatment. Therefore, a DXR concentration of 0.1 μM was considered sublethal, and the concentrations of 0.5 and 1 μM were considered toxic, although the latter ones were still not approaching IC_90_. We observed differential effects between motility and morphology characteristics in sublethal and toxic doses, which were probably the consequences of different cellular responses under these concentrations. The changes in morphology and motility observed during the treatment with a sublethal dose were likely caused solely by DXR since the dose does not lead to massive cell death (as estimated by Alamar blue assay with 94% of viable cells after 48 h treatment and IC_50_, Figure 4b). However, under 0.5 and 1 μM concentrations, apoptotic events were observed, which likely contributed to the observed phenotypes. Therefore, DXR treatment significantly affected cellular motility and morphology, probably associated with induced TTP and *CTTN* levels, but also with apoptotic events in case of the treatment with toxic concentration.

## 3 DISCUSSION

Despite intensive investigations of the role of TTP in cancer progression and inhibition of invasion, our knowledge about its impact on basic mechanisms of cell motility and invasiveness remains limited. Here, we investigated if TTP can affect components of the cytoskeleton involved both in cell movement and invasion, such as actin nucleation factors N‐WASP (*WASL*) and cortactin (*CTTN*), WIP (*WIPF1*), and scaffold proteins TKS4 and TKS5 (*SH3PXD2B* and *SH3PXD2A*, respectively). Our findings show that invasive MDA‐MB‐231 cells with ectopic TTP expression exhibited 1.3‐fold increased mRNA levels of *SH3PXD2B* and nearly 2‐fold decreased mRNA levels of *SH3PXD2A* and *CTTN*, which may explain significant changes in cellular motility and morphology compared to wild‐type cells. Previously, Bryce and colleagues observed decreased cell migration and invasion in cortactin‐deficient cells, which exhibited a selective defect in the stability of lamellipodia protrusions and reduced levels of free barbed ends of actin filaments (Bryce et al., 2005). We suggest that the decrease of motility and the ability to directed movements and invasion observed in the current study was caused by cells' inability to efficiently polymerize actin filaments due to a lack of *SH3PXD2A* and *CTTN*. Since Arp2/3 complex itself is a weak nucleation factor and its direct interaction with cortactin stimulates its polymerization activity, and TKS5 is required for the assembly of remodeling complex, simultaneous decrease of *SH3PXD2A* and *CTTN* could result in both insufficient recruitment of the Arp2/3 complex and its insufficient activity, leading to decreased actin polymerization capacity. Together with a decrease in cortactin levels, we observed fewer F‐actin fibers, and their bundling compared to those in normal cells and the lack of lamellipodia. Since cortactin is necessary for actin filament branching, a decrease of its mRNA level in TTP overexpressing cells may contribute to the changes in actin morphology, including the bundling of actin filaments (T. Liu et al., 2024). Interestingly, we observed the emergence of thick F‐actin filaments in the cortical region of the cellular membrane and the disappearance of stress fibers. The role of TTP in the organization of cortical actin caps was previously observed in mouse oocytes (T. Liu et al., 2024). Moreover, although the average F‐actin intensity of the cells with ectopic TTP expression increased, indicating denser F‐actin staining, the total F‐actin signal per cell slightly but significantly decreased, reflecting smaller cell size, but possibly also weaker actin polymerization capacity. Similar actin morphology, including the emergence of F‐actin‐rich spots, was described in the cells treated with cytochalasin B, an actin polymerization inhibitor, which supports our hypothesis regarding impaired actin polymerization (Elosegui‐Artola et al., 2014; Lambert et al., 2023). The smaller size of the TTP‐expressing cells may be explained by the lack of extended lamellipodia, which are present in the control cells, although both cell lines showed a “cobblestone” shape typical for MDA‐MB‐231 cells (Franchi et al., 2020). Also, we observed the increase of *SH3PXD2B* with the decrease of motility and invasion. Several studies indicate that it can both promote and inhibit cell migration. For example, Bogel and colleagues show that in EGF‐treated cells, the knock‐down of TKS4 led to significant inhibition of HeLa cell migration and invasion, indicating its role in promoting motility (Bögel et al., 2012). However, another study shows that in primary human umbilical vein endothelial cells, the absence of TKS4 led to decreased motility (Mehes et al., 2019). We suggest that more research is needed to clarify the role of TKS4 in motility and whether its effect is cell type‐specific or if it may depend on other factors (e.g., differential expression of its isoforms).

We also investigated the effects of a chemotherapeutic drug, doxorubicin, widely used in TNBC, on expression levels of target genes, as well as on cell morphology and motility. In this study, we treated the MDA‐MB‐231 cells (corresponding to TNBC) with clinically relevant concentrations of doxorubicin (up to 1 μM) for 48 h. We observed that even a low concentration of 0.1 μM DXR led to a significant increase in TTP expression, reaching 15‐fold under 1.0 μM DXR treatment. Previously, Lee and colleagues showed that MDA‐MB‐231 cells treated for 24 h with DXR did not demonstrate significant changes in TTP expression. However, in many other cell lines, including poorly invasive MCF7 cells, which possess wild‐type p53, they observed a p53‐dependent increase of TTP expression (J. Y. Lee et al., 2013). We suggest that 24 h might not be enough for the MDA‐MB‐231 cells to develop the response since the line is characterized to have mutated p53 and thus impaired response to DNA damage. As our data show, cells indeed demonstrated the expected increase of TTP levels after 48 h. Given that increased TTP expression in ecTTP cells led to significant changes in target cytoskeleton‐associated mRNA levels, we presumed that DXR‐induced TTP might lead to similar alterations in target mRNA levels and cellular phenotype. However, our results did not support this hypothesis, as the treatment resulted in an overall increase in all target mRNAs, likely indicating a generalized response to DNA damage. Specific targets, such as *SH3PXD2A*, *SH3PXD2B*, and *WIPF*, exhibited a slight increase in mRNA levels at 0.1 and 0.5 μM of DXR but remained unchanged at the 1 μM DXR concentration. Interestingly, our data show that DXR induced a sustainable level of cortactin mRNA. Discrepancies between target mRNA levels in doxorubicin‐treated and ecTTP cells might be explained by the fact that DXR activates and disrupts multiple signaling pathways, which may obscure the effects of TTP. Another factor that could contribute to such target mRNA levels is that TTP is known to downregulate its own mRNA, a process expected to occur under DXR treatment as well (Tchen et al., 2004). However, TTP introduced to the cells to facilitate ectopic expression lacks three out of four binding sites for its product, disrupting autoregulation. This likely resulted in a twofold higher TTP level in ecTTP cells compared to DXR‐treated wild‐type cells and could potentially affect target mRNA levels.

The treatment also affected cell motility and morphology: DXR decreased motility in a dose‐dependent manner starting from 0.5 μM concentration, as well as led to significant changes in cytoskeletal morphology and increased cell size. Surprisingly, the most dramatic effect of DXR on cell area and number of actin filaments per cell was observed at the concentration of 0.1 μM. As described in the literature, DXR affects actin polymerization by reducing fiber size, growth, and amount of fibers in steady‐state (Colombo et al., 1988; Colombo & Milzani, 1988), as well as it might induce the formation of giant cells, which supports our data (Eddy et al., 2017). There is also evidence that DXR induces cortical F‐actin formation associated with cortical translocation of p‐MLC from central stress fibers (Wei et al., 2015). However, the reported data on the DXR effect on cell motility are inconsistent: some studies claim it inhibits motility (Effat et al., 2024; Hernandes et al., 2023; Wang et al., 2019). However, other studies show motility increase (Hernandes et al., 2023; C.‐L. Liu et al., 2019; Mohammed et al., 2021). We suggest that this differential effect depends on the cells' sensitivity to the drug. Many studies showing DXR activating effect on cell motility use the term “sublethal dose,” typically administering doses between 25% and 50% of the IC_50_ calculated for each specific culture. In our study, DXR exceeding IC_50_ (0.19 μM) decreased cell motility. In contrast, a sublethal concentration of 0.1 μM did not affect displacement but did decrease the confinement ratio and median speed. Thus, we suggest the difference between morphological phenotypes at 0.1 μM versus 0.5 μM and 1.0 μM concentrations of DXR might be related to discrepancies in cellular physiology while exposed to the sublethal versus toxic dose.

The data on DXR‐induced TTP expression obtained in the current study also raise concern about its use as a positive prognostic marker in TNBC patients. BC is a highly heterogeneous malignancy characterized by the presence or absence of estrogen, progesterone, and HER2/neu receptors, as well as a variety of other marker molecules. The therapeutic approaches include not only hormonal therapy but also chemotherapeutics. In the case of TNBC, which is characterized by the absence of all three hormone receptors mentioned above, there is only one FDA‐approved treatment option—chemotherapy, and doxorubicin is one of the most widely used (Waks & Winer, 2019). Since many studies consider high TTP expression as a positive prognostic marker in BC patients, we question whether it may be used if the patient was administrated with doxorubicin. If doxorubicin induces TTP expression in vivo, it will be challenging to conclude whether the high TTP expression level reflects the patient's physiological condition or was induced by the therapy itself. Considering the p53‐dependent mechanism of TTP induction described by J. Y. Lee et al., (2013), it is also possible that TTP could be induced by other DNA‐damaging chemotherapeutics, such as cisplatin, its derivatives, or methotrexate. Additionally, since DXR is known to be absorbed by tissues, the duration of its effects on TTP expression post‐administration, if it occurs in vivo, is uncertain. Therefore, we recommend further studies to investigate whether doxorubicin administration leads to a significant increase in TTP expression in vivo, if it is TNBC‐specific, for how long the effect persists, and if it affects its prognostic reliability, since, in our opinion, it may be of use for the development of reliable personalized medicine approaches and diagnostics.

## 4 CONCLUSION

BC remains a significant global health concern, demanding further exploration to improve prevention, detection, and treatment strategies, particularly regarding metastasis. Therefore, understanding the molecular mechanisms governing cell motility and invasion is crucial. Here, we show that ectopic TTP expression significantly impairs cell motility via dysregulation of cytoskeleton‐associated genes. Although it is not known if TTP directly binds to target gene mRNAs and regulates them in a straightforward way, we suggest our findings open a promising research field, contribute to existing knowledge, and widen it. Moreover, our findings contribute to understanding the effects of doxorubicin on cell expression profiles.

## 5 LIMITATIONS

Although in the current study we observed significant changes in *CTTN*, *SH3PXD2A*, and *SH3PXD2D* mRNA levels, as well as prominent changes in the phenotype of the ecTTP cells, such changes in mRNA levels may not necessarily lead to the changes in corresponding protein levels. More studies are needed to investigate if TTP overexpression affects protein levels of CTTN, TKS5, and TKS4. In addition, although doxorubicin significantly increased TTP expression in TNBC cells, such an effect may not occur in vivo. We suggest it would be of use to explore such an effect clinically to evaluate the reliability of TTP as a positive prognostic marker in TNBC patients. A more detailed study of the effect of TTP and DXR on the actin regulatory network is needed, including the analysis of cells using higher magnification imaging, as well as efficient exclusion of dead and dying cells in the experiments utilizing DXR.

## 6 MATERIALS AND METHODS

**Cell culture and treatment**: MDA‐MB‐231 (ATCC HTB‐26) BC cell line kindly provided by Prof. Jiri Bartek (Copenhagen) was used in the present study. The cells were cultivated in Dulbecco's modified Eagle medium with high glucose content (Gibco, #11965092) supplemented with 10% fetal bovine serum and penicillin/streptomycin at 37°C, 5% CO_2_ in a humidified incubator. When they reached about 70%–80% confluence, they were treated with 0.1, 0.5, or 1.0 μM of doxorubicin (Sigma, D1515) and incubated for 48 h, subsequently trypsinized and washed twice with cold phosphate buffered saline. After that, the content of each 10‐cm Petri dish was divided into two microcentrifuge tubes, one for RNA isolation and one for protein lysate preparation for further analysis by RT‐qPCR and Western blotting, respectively. All the steps after trypsinization were kept on ice. All the data were obtained from at least two independent experiments performed at least in triplicate.

**Stable cell line generation**: The cells were transfected with plasmid construction pcDNA4‐TO‐TTP, obtained previously in our laboratory, previously using Lipofectamine 3000 reagent (Invitrogen, L3000001) according to manufacturer's recommendations. After 24 h of transfection, the medium was aspirated, and the cells were supplied with fresh medium containing 200 μg/mL zeocin (InvivoGen) for 3 weeks. Fresh medium with a new dose of zeocin was replenished every other day. After all nonresistant cells died, resistant cells were seeded at 96‐well plates, 1 cell per well, for further selection. The presence of overexpression of TTP was verified via Western blotting analysis.

**Transwell invasion assay:** MDA‐MB‐231 WT and ecTTP cells were grown to approximately 70% confluence and starved in a serum‐free medium for 24 h before the experiment. On the day of the experiment, the cells were seeded to the transwell insert (Corning, #3422) precoated with Geltrex (Thermo Fisher, #A1413201) at a density 100,000 per well in a serum‐free medium. Ten percent FBS was used as a chemoattractant. After 20 h of incubation, the cells were washed with PBS, fixed in 3.8% formaldehyde, and stained with 0.2% crystal violet. The cells on the inner part of the permeable membrane were removed with a cotton swab. The images of the membranes were taken using an Axio Zoom microscope (Zeiss), and further processed with ImageJ software. The invasion was calculated as an invaded area.

**Western blotting analysis**: Protein lysates were prepared in RIPA buffer supplemented with EDTA‐free Halt protease and phosphatase inhibitors cocktail (Thermo Scientific, #78425). Protein concentrations were measured using BCA assay (Pierce, #23225), and an equal amount of protein (e.g., 10 μg) was loaded on NuPAGE Bis‐Tris 4%–12% gel (Invitrogen, NP0322BOX). After electrophoresis, the samples were transferred to nitrocellulose membrane using Trans‐Blot Turbo system (BioRad, #1704270). The membrane was stained with Ponceau S total protein staining solution (Thermo Fisher, #A40000279), followed by signal documentation. After that, the membrane was washed three times with Millipore water, followed by blocking with 5% BSA for 1 h, incubation overnight with primary antibody (anti‐TTP, Cell Signaling, #71632, 1:1000) and then secondary antibody (anti‐rabbit, Jackson ImmunoResearch, AB_10015282, 1:15,000). The signal was developed using an ECL detection reagent (Amersham, RPN2105) and documented with ChemiDoc device (BioRad). After imaging, the membrane was washed three times with TBST and incubated with 30% hydrogen peroxide to inhibit peroxidase activity (described by Sennepin et al., 2009). Membrane was further washed with TBST and incubated with an anti‐tubulin primary antibody (Sigma, T4026, 1:10,000), followed by incubation with a secondary antibody (anti‐mouse Jackson ImmunoResearch, AB_10015289, 1:20,000) and further signal development and documentation. Signal intensity was calculated using ImageLab software (BioRad).

**Cytotoxicity and metabolic activity assay**: To evaluate viability wild‐type and transformed MDA‐MB‐231 cells were seeded in 96‐well plates (10,000 cells/well) and left to sit overnight. Then, the medium was replenished with the one containing 10% Alamar Blue reagent (Invitrogen, DAL1100) and incubated in a humidified CO_2_ incubator for 2 h. Subsequently, absorbance was measured using a microplate reader; then, the cells were washed with PBS and replenished with the regular medium. After 48 h, the procedure was repeated. For the calculation of cell proliferation, the first measurement was used as a control. To measure doxorubicin cytotoxicity, the cells were seeded in 96‐well plates (10,000 cells/well), let sit overnight, and subsequently treated with different concentrations of doxorubicin. After 72 h of incubation, the cells were washed with PBS, and a fresh medium containing 10% Alamar Blue reagent was added. After 2 h of incubation, absorbance was measured using a microplate reader. Untreated cells were used as a control.

**RT‐qPCR**: Total RNA was isolated using an RNAeasy mini Plus kit (Qiagene, #74134). An equal amount of RNA (20 ng) was loaded to each reaction. RT‐qPCR was performed using LUNA Universal One‐step RT‐qPCR mix (NEB, E3005). Primer efficiency was calculated for each primer pair (a list of primers used with efficiency values may be found in Table S2). Melting curve analysis was performed for each sample. The following formula was used to calculate PCR results:

$$\mathrm{Exp}={\left(E_{\mathrm{GOI}}\right)}^{∆\mathrm{Ct}\left(\mathrm{GOI}\right)}/{\left(E_{\mathrm{HKG}}\right)}^{∆\mathrm{Ct}\left(\mathrm{HKG}\right)},$$

where Exp is expression, *E*_GOI_ is primers efficiency for the gene of interest, ∆Ct_(GOI)_ is ∆Ct for the gene of interest, *E*_HKG_ is primers efficiency for the housekeeping gene, ∆Ct_(HKG)_ is ∆Ct for the housekeeping gene.

**Confocal microscopy and cell tracking**: For tracking of stable transfectants cell movement, cells were seeded at a density of 22,000 cells/cm^2^ in Nunc™ Lab‐Tek™ Chambered Coverglass (1.0 borosilicate glass, #155383) and labeled with SiR‐DNA (Spirochrome, 251SC007) and SPY555‐actin (Spirochrome, SC202) according to manufacturer's recommendations. Images were acquired every 15 min for 24 h. For tracking, a confocal microscope Leica TCS SP5 equipped with HCX PL APO CS 10.0 × 0.40 DRY UV objective was employed. Cells were incubated at 36°C with 5% CO_2_. For scanning, the pinhole was open to 2 Airy units, the samples were bidirectionally scanned at 400 Hz speed and 1024 × 1024 pixel image resolution. For excitation of SPY555‐actin and SiR‐DNA laser lines 561 and 633 nm, respectively, and for detection PMT detector and hybrid detector, at 570–620 and 650–700 nm, respectively, were employed. The bright field was detected using PMT trans. For preprocessing of the images, maximal projection in Z was computed, fluorescence bleed‐through was compensated by subtracting the SPY555‐actin channel from the SiR‐DNA channel and Enhance Local Contrast (CLAHE) plugin of ImageJ was applied. Then, the nuclei of the cells were tracked by StarDist Versatile (fluorescent nuclei) model in the TrackMate plugin of ImageJ/Fiji using a LAP tracker (linking 20 μm, gap closing 20 μm, one frame, track segment splitting 10 μm) (Ershov et al., 2022; Schindelin et al., 2012; Schmidt et al., 2018). Nuclei tracked for less than 2/3 of the experiment duration were not analyzed. The tracking was performed in biological duplicates; at least 500 nuclei were tracked in each replicate.

For the analysis of doxorubicins effect on cell motility, MDA‐MB‐231 cells were seeded at a density of 44,000 cells/cm^2^ in Nunc™ Lab‐Tek™ Chambered Coverglass, let sit overnight and treated for 48 h with 0.1, 0.5, and 1.0 μM doxorubicin. After incubation, the medium was replenished, and the cells were labeled with SiR‐DNA. Further analysis was performed as described above.

For the analysis of actin cytoskeleton, cells were seeded at 22,000 cells/cm^2^ in Nunc Lab‐Tek II Chamber Slide™, fixed with 3.8% formaldehyde in PBS, stained by Phalloidin‐iFluor 488 reagent (Abcam, #ab176753) according to manufacturer's protocol and mounted in laboratory‐made Mowiol with 0.25 μg/mL DAPI for DNA staining. Imaging was performed using a confocal microscope Leica STELLARIS 8, which was equipped with HC PL APO CS2 40 × 1.25 GLYC objective. For scanning, the pinhole was closed to one Airy unit, and the sample was unidirectionally scanned at 400 Hz speed with three‐line averaging and 1024 × 1024 pixel image resolution. For excitation of DAPI and Phalloidin‐iFluor, 405 diode laser and 493 nm laser line of WLL were used for excitation, respectively, and for detection hybrid detectors at 425–500 and 500–550 nm, respectively, were employed. For preprocessing of the images, maximal projection in Z was computed. For the analysis of actin filaments, FilamentSensor 2.0 was utilized with default settings (Hauke et al., 2023). For the analysis of cell morphology, the preprocessed images were blurred by a Gaussian filter with a 2 μm radius and used for segmentation by CellPose3 using a cyto3 model, diameter 150 px, flow threshold 0.6, blurred phalloidin as a cytoplasmic channel, and blurred DAPI compensated for fluorescence bleed‐through as a nuclear channel (Stringer & Pachitariu, 2024). Found masks were then used to measure cell morphology using original preprocessed images in ImageJ.

For the analysis of doxorubicin effect on morphology, MDA‐MB‐231 cells were seeded at a density of 44,000 cells/cm^2^ in Nunc™ Lab‐Tek™ Chambered Coverglass, let sit overnight and treated for 48 h with 0.1, 0.5, and 1.0 μM doxorubicin. After incubation, the cells were fixed with 3.8% formaldehyde in PBS, stained by Phalloidin‐iFluor 647 reagent (Abcam, #ab176753) according to the manufacturer's protocol to avoid doxorubicin excitation, and mounted in laboratory‐made Mowiol with 0.25 μg/mL DAPI for DNA staining. Further analysis was performed as described above.

**Statistical analysis**: Statistical analysis was performed with Origin 2021b and PrismGraphPad 9.5 software. All the data were checked for normality. Parametric data were analyzed by analysis of variance (ANOVA) for multiple group comparison and a two‐tailed *t*‐test for pairwise comparison. Nonparametric data were analyzed using Kruskal‐Wallis ANOVA for multiple group comparison and Mann–Whitney's test for pairwise comparison. A value of 0.05 was set as a threshold of significance. All the graphs are presented as mean ± standard deviation. *p < 0.05; **p < 0.01; ***p < 0.001; ****p < 0.0001.

## AUTHOR CONTRIBUTIONS

*Manuscript writing*: A.H. and J.N. *Cell culture and treatment, gene expression, cytotoxicity and viability experiments, stable cell line generation*: A.H. *Invasion assay*: A.H. and J.N. *Western blotting analysis*: A.H. and D.S. *Confocal microscopy and cell tracking*: A.H., J.N., and M.V. *Statistical analysis*: A.H. and J.N. *Experimental design, conceptualization, and validation*: A.H., S.K., and Z.H.

## FUNDING INFORMATION

This study was supported by the following grants: Grant Agency of the Czech Republic (Project 24‐11357S); Institute of Molecular Genetics of the Czech Academy of Sciences, Project RVO 68378050; European Regional Development Fund, CZ.02.01.01/00/22_008/0004562 Project “Excellence in Regenerative Medicine”); Akademie Ved Ceske Republiky—Strategy AV21 (VP29) Toward Precision Medicine and Gene Therapy; A.H. was supported by “Researchers at risk” fellowship of the Czech Academy of Sciences (RRFU‐22‐22, 05/RISK/EO/22).

## CONFLICT OF INTEREST STATEMENT

The authors declare no conflicts of interest.

## Supporting information


**Data S1.** Supporting Information.

## Data Availability

The data that support the findings of this study are available at https://doi.org/10.5281/zenodo.13768704.
