# Supplementary material for: Tristetraprolin affects invasion‐associated genes expression and cell motility in triple‐negative breast cancer model
Source: Cytoskeleton (Hoboken). 2024 Sep 25;82(5):311–26. doi: 10.1002/cm.21934 (PMC12063522; doi:10.1002/cm.21934)
Supplement: Supplementary file 1 — Data S1. Supporting Information. [file CM-82-311-s001.zip › cm21934-sup-0001-supinfo1.docx]

**SUPPORTING INFORMATION**

**Tristetraprolin Affects Invasion-Associated Genes Expression and Cell Motility in Triple-Negative Breast Cancer Model**

**Anastasiia Hubiernatorova^*^, Josef Novak, Michaela Vaskovicova, David Sekac, Serhii Kropyvko, Zdenek Hodny**

**Anastasiia Hubiernatorova,* anastasiia.hubiernatorova@img.cas.cz; april.scorpionen@gmail.com

**Table S1.** In silico analysis of 3’UTRs of genes involved in migration and invasion

| 1 | Gene^[[1]](#footnote-1)^*^[[2]](#footnote-2)^ | | Arp2 | Arp3 | Arpc3 | Arpc2 | Arpc4 | Arpc1B | WASP | N-WASP | WASF1 | WASF2 (1)* | WASF2  (2)* | WASF3 |
| --- | --- | --- | --- | --- | --- | --- | --- | --- | --- | --- | --- | --- | --- | --- |
| 2 | *3’UTR length, nt* | | *2530* | *4106* | *442* | *442* | *907* | *312* | *278* | *2592* | *713* | *3980* | *4115* | *3116* |
| 3 | *Regulatory element* | ARE |  |  |  |  |  |  |  |  |  |  |  |  |
| 4 |  | Brd-box |  |  |  |  |  |  |  |  |  |  |  |  |
| 5 |  | C-Rich stability element |  |  |  |  |  |  |  |  |  |  |  |  |
| 6 |  | Grb-box |  |  |  |  |  |  |  |  |  |  |  |  |
| 7 |  | GU-rich element |  |  |  |  |  |  |  |  |  |  |  |  |
| 8 |  | GY-Box |  |  |  |  |  |  |  |  |  |  |  |  |
| 9 |  | K-Box |  |  |  |  |  |  |  |  |  |  |  |  |
| 10 |  | Musashi |  |  |  |  |  |  |  |  |  |  |  |  |
| 11 |  | Pumilio binding element |  |  |  |  |  |  |  |  |  |  |  |  |
| 12 |  | SECIS1 |  |  |  |  |  |  |  |  |  |  |  |  |
| 13 |  | UNR binding site |  |  |  |  |  |  |  |  |  |  |  |  |

| Source |  | Scan for Motifs |  | RegRNA 2.0 |  | Both |
| --- | --- | --- | --- | --- | --- | --- |

**Table S1** (continuation)

| 1^[[3]](#footnote-3)^* | Cdc42  (1,3) | Cdc42  (2)* | SH3PXD2A | SH3PXD2B | RhoG | RhoD | RhoB | RhoF | RhoQ | Formin1  (1,2)* | Formin1  (3)* | Formin2 | BAIAP2 |
| --- | --- | --- | --- | --- | --- | --- | --- | --- | --- | --- | --- | --- | --- |
| 2 | *1440* | *788* | *8149* | *4870* | *599* | *433* | *1402* | *1761* | *3606* | *8783* | *1494* | *1047* | *1514* |
| 3 |  |  |  |  |  |  |  |  |  |  |  |  |  |
| 4 |  |  |  |  |  |  |  |  |  |  |  |  |  |
| 5 |  |  |  |  |  |  |  |  |  |  |  |  |  |
| 6 |  |  |  |  |  |  |  |  |  |  |  |  |  |
| 7 |  |  |  |  |  |  |  |  |  |  |  |  |  |
| 8 |  |  |  |  |  |  |  |  |  |  |  |  |  |
| 9 |  |  |  |  |  |  |  |  |  |  |  |  |  |
| 10 |  |  |  |  |  |  |  |  |  |  |  |  |  |
| 11 |  |  |  |  |  |  |  |  |  |  |  |  |  |
| 12 |  |  |  |  |  |  |  |  |  |  |  |  |  |
| 13 |  |  |  |  |  |  |  |  |  |  |  |  |  |

| Source |  | Scan for Motifs |  | RegRNA 2.0 |  | Both |
| --- | --- | --- | --- | --- | --- | --- |

**Table S1** (continuation)

| 1^[[4]](#footnote-4)^* | VASP | FNBP1 | TRIP10  (1,2)* | TRIP10  (3)* | FNBP1L  (1)* | FNBP1L  (2,3)* | CFL1 | CFL2 | FCHO1 | FCHO2 | EPS15 | ITSN1  (S)* | ITSN1  (L)* | ITSN2  (S)* | ITSN2  (L)* |
| --- | --- | --- | --- | --- | --- | --- | --- | --- | --- | --- | --- | --- | --- | --- | --- |
| 2 | *813* | *3385* | *315* | *200* | *3564* | *2273* | *525* | *2483* | *268* | *2431* | *2437* | *1510* | *11561* | *589* | *762* |
| 3 |  |  |  |  |  |  |  |  |  |  |  |  |  |  |  |
| 4 |  |  |  |  |  |  |  |  |  |  |  |  |  |  |  |
| 5 |  |  |  |  |  |  |  |  |  |  |  |  |  |  |  |
| 6 |  |  |  |  |  |  |  |  |  |  |  |  |  |  |  |
| 7 |  |  |  |  |  |  |  |  |  |  |  |  |  |  |  |
| 8 |  |  |  |  |  |  |  |  |  |  |  |  |  |  |  |
| 9 |  |  |  |  |  |  |  |  |  |  |  |  |  |  |  |
| 10 |  |  |  |  |  |  |  |  |  |  |  |  |  |  |  |
| 11 |  |  |  |  |  |  |  |  |  |  |  |  |  |  |  |
| 12 |  |  |  |  |  |  |  |  |  |  |  |  |  |  |  |
| 13 |  |  |  |  |  |  |  |  |  |  |  |  |  |  |  |

**Figure S1**. Effect of TTP ectopic expression on MDA-MB-231 movement and morphology


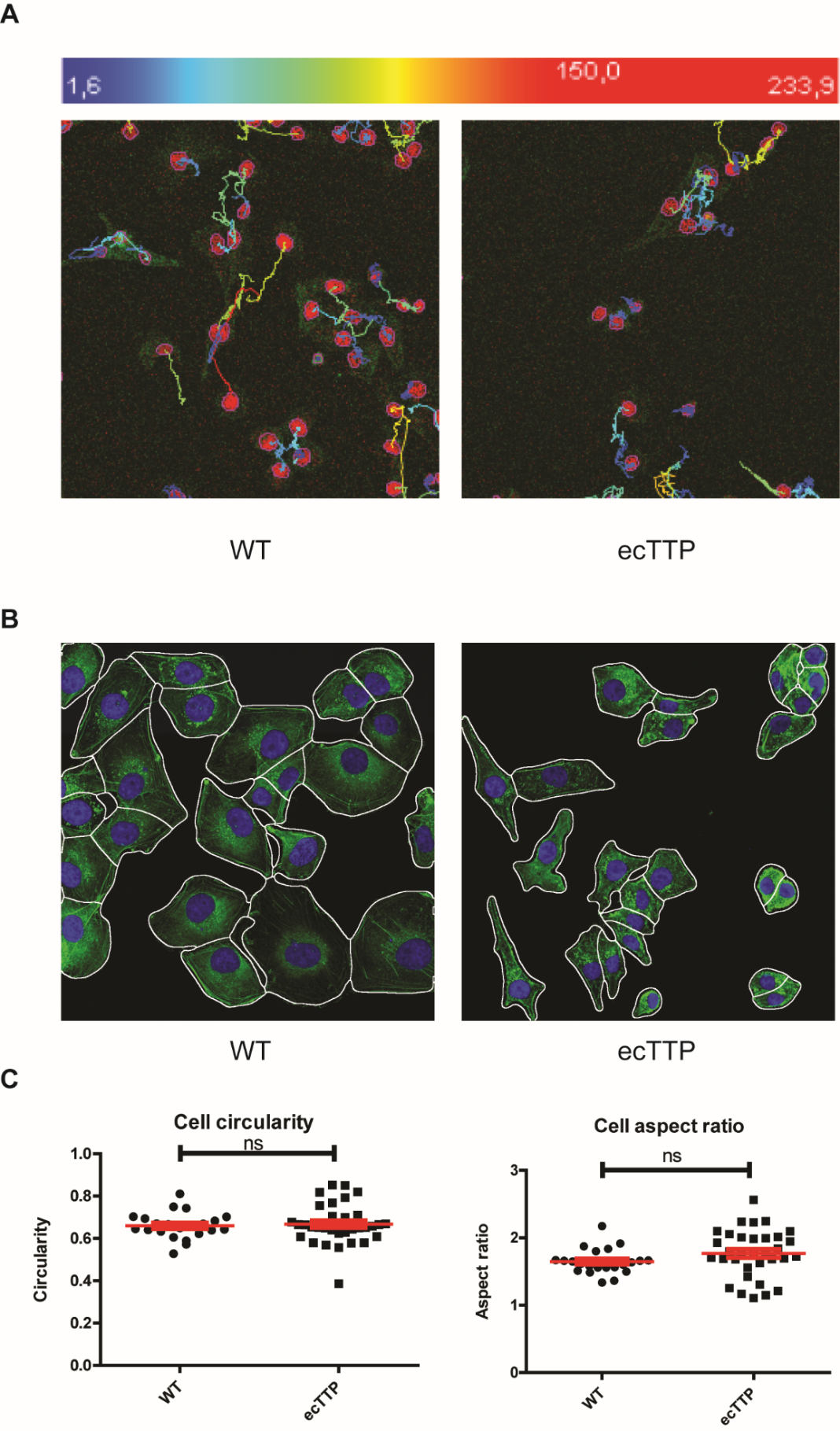


A) Representative images (400×400 µm regions) showing trajectories of the tracked nuclei. Color of the tracks represents displacement of the nuclei according to the calibration bar shown above the images, all nuclei with displacement above 150 µm are shown in red. Nuclei are shown in red (SiR-DNA), F-actin in green (SPY555-actin). B) Outlies of representative CellPose segmentation mask (white) used for quantification of cellular morphology. C) Effects of TTP ectopic expression on cellular circularity and aspect ratio.

**Figure S2.** Representative images of the transwell invasion assay

**
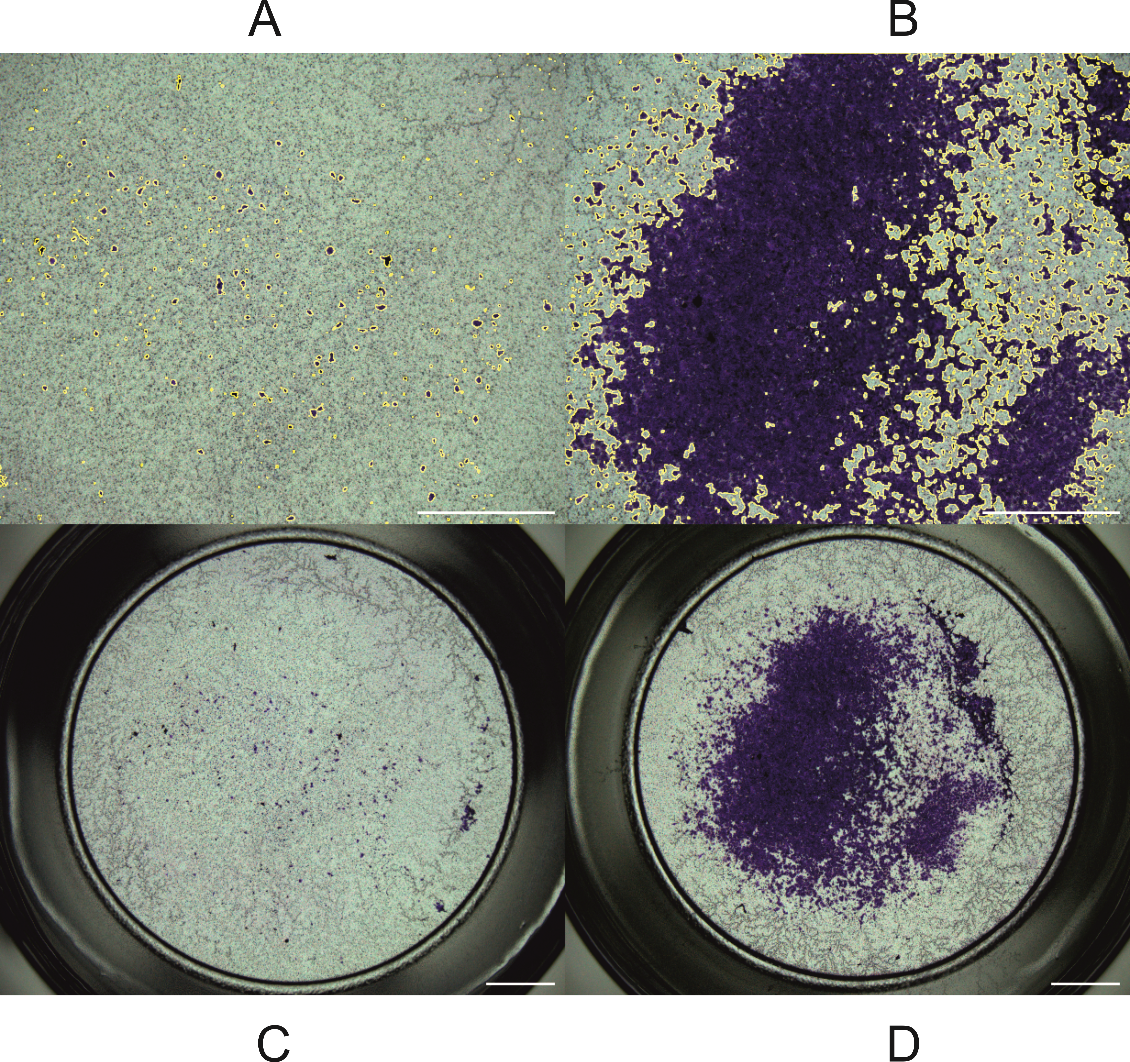
**

A, B) Representative images of ImageJ area segmentation of ecTTP and WT MDA-MB-231 cells, respectively. C, D) Representative images of the whole membrane of ecTTP and WT MDA-MB-231 cells, respectively. Scale bar, 1000 µm.

**Figure S3.** Effect of doxorubicin on MDA-MB-231 movement and morphology.

**
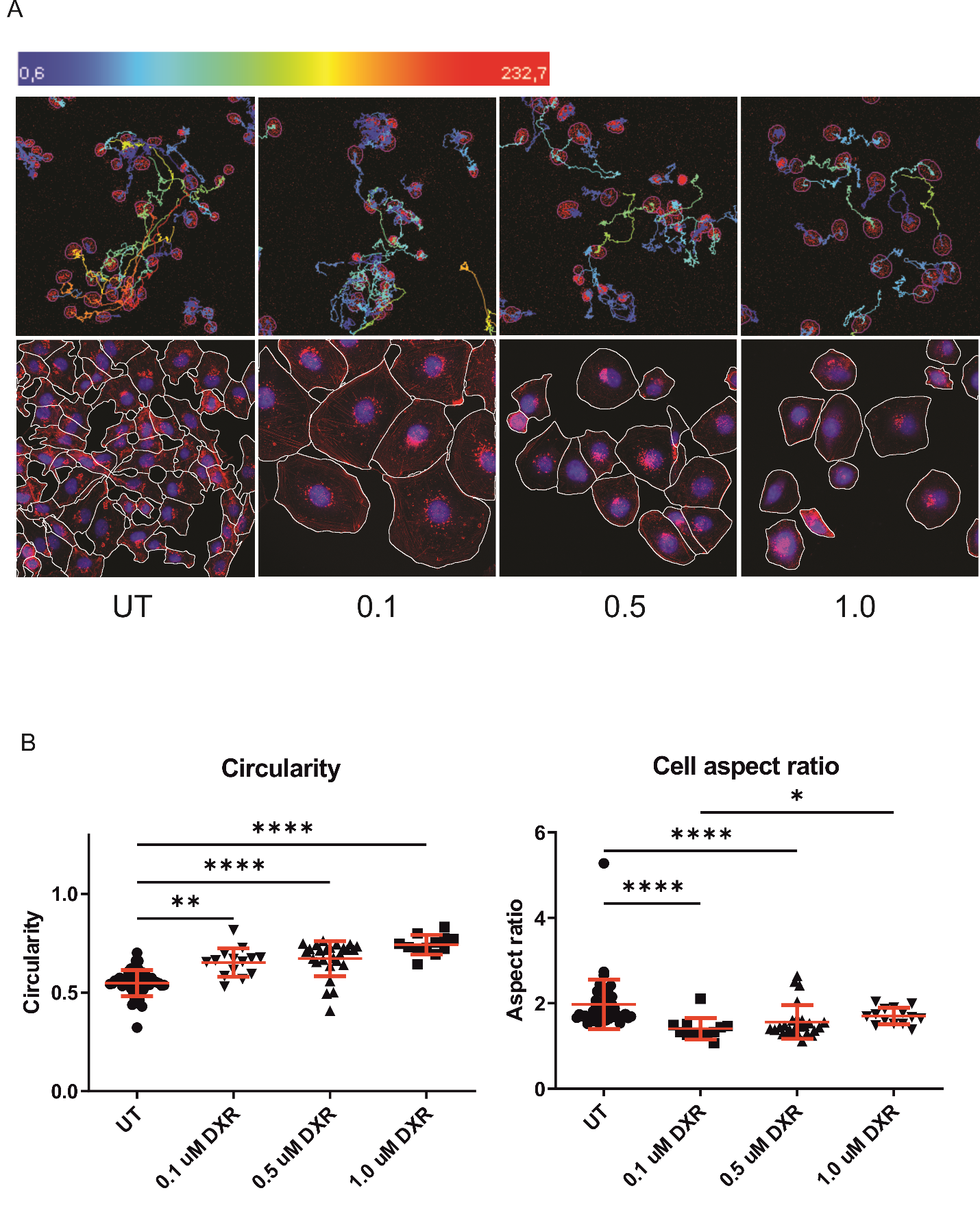
**

A) Upper panel **–** representative images (400×400 µm regions) showing trajectories of the tracked nuclei. Color of the tracks represents displacement of the nuclei according to the calibration bar shown above the images, all nuclei with displacement above 200 µm are shown in red. Nuclei are shown in red (SiR-DNA); bottom panel – outlies of representative CellPose segmentation masks (white) used for quantification of cellular morphology. Note that in Figure 5 actin is visualized in green.; B) Effects of doxorubicin on circularity and aspect ratio in treated cells in comparison to control cells. UT – untreated cells; 0.1, 0.5, and 1.0 µM concentration of doxorubicin. Graphs show mean ± standard deviation. *p<0.05, **p<0.01, ***p<0.001, ****p<0.0001.

**Table S2.** Sequences and efficiencies of primers used

| **Primer pair** | **Sequence (5’-3’)** | **Efficiency** |
| --- | --- | --- |
| For-ZFP36 (TTP)  Rev-ZFP36 (TTP) | TCTTCGAGGCGGTTTTT  TGCGATTGAAGATGGGGAGTC | 93.5% |
| For-SH3PXD2A  Rev-SH3PXD2A | CGAACCTACGGACAAGACCTC  CGTGGCTTTGGCAGTTGGAA | 93.78% |
| For-SH3PXD2B  Rev-SH3PXD2B | GGCTGTCAAACGCCTGATAC  GGTTTGGTCACCCCCAGATTT | 100.82% |
| For-WIPF1  Rev-WIPF1 | ACGGCCAACAGGGATAATGAT  GGTTTCGCAGATGTGGATCTT | 102.13% |
| For-WASL  Rev-WASL | GAACGAGTCCCTCTTCACTTT  TTCCGATCTGCTGCATATAACT | 105.8% |
| For-CTTN  Rev-CTTN | GCTTTGAGTATCAAGGCAAAACG  CCAACGGCACATTTGTCTTGT | 90.13% |
| For-GAPDH  Rev- GAPDH | AGCCACATCGCTCAGACAC  GCCCAATACGACCAAATCC | 101.3% |

1. * data on genes with distinct 3’UTRs are presented separately for each isoform (number is indicated in brackets) [↑](#footnote-ref-1)
2. [↑](#footnote-ref-2)
3. * data on genes with distinct 3’UTRs are presented separately for each isoform (number is indicated in brackets) [↑](#footnote-ref-3)
4. * data on genes with distinct 3’UTRs are presented separately for each isoform (number is indicated in brackets) [↑](#footnote-ref-4)
